# Small-area analyses of bone cancer diagnosed in Great Britain provide clues to aetiology

**DOI:** 10.1186/1471-2407-12-270

**Published:** 2012-06-27

**Authors:** Richard J Q McNally, Karen Blakey, Roger C Parslow, Peter W James, Basilio Gómez Pozo, Charles Stiller, Tim J Vincent, Paul Norman, Patricia A McKinney, Michael F Murphy, Alan W Craft, Richard G Feltbower

**Affiliations:** 1 Institute of Health and Society, Newcastle University, England, UK; 2 Centre for Epidemiology & Biostatistics, University of Leeds, England, UK; 3 Childhood Cancer Research Group, Department of Paediatrics, University of Oxford, England, UK; 4 School of Geography, University of Leeds, England, UK; 5 Northern Institute of Cancer Research, Newcastle University, England, UK; 6 Institute of Health and Society, Newcastle University, Sir James Spence Institute, Royal Victoria Infirmary, Queen Victoria Road Newcastle upon Tyne, NE1 4LP, England, UK

## Abstract

****Background**:**

The aetiology of bone cancers is poorly understood. This study examined geographical patterning in incidence of primary bone cancers diagnosed in 0–49 year olds in Great Britain during 1980–2005 to provide information on factors linked with disease development. We investigated putative associations with deprivation and population density.

****Methods**:**

Data on osteosarcoma and Ewing sarcoma were obtained from national population-based registries. Negative binomial regression was used to examine the relationship between incidence rates and the Townsend deprivation score (and its component variables) and small-area population density.

****Results**:**

The study analyzed 2566 osteosarcoma and 1650 Ewing sarcoma cases. For females with osteosarcoma, statistically significant decreased risk was associated with higher levels of deprivation (relative risk [RR] per unit increase in deprivation score = 0.969; 95% confidence interval [CI] 0.946–0.993). For all Ewing sarcoma combined, statistically significant decreased risk was associated with greater area-level population density and higher levels of non-car ownership (RR per person per hectare increase = 0.984; 95% CI 0.976–0.993, RR per 1% increase in non-car ownership = 0.994; 95% CI 0.991–0.998).

****Conclusions**:**

Higher incidence of osteosarcoma was observed for females in areas with lower deprivation levels indicating increased risk is linked to some aspect of affluent living. Higher incidence of Ewing sarcoma occurred in areas of low population density and where more people owned cars, both characteristic of rural environments. The study adds substantially to evidence associating Ewing sarcoma risk with rural environmental exposures. Putative risk factors include agricultural exposures, such as pesticides and zoonotic agents.

## **Background**

The exact aetiology of bone cancer is poorly understood. In the UK, at all ages, the age-standardized rate (to the world population) is 8 per 1,000,000 persons per year for males and 6 per 1,000,000 persons per year for females [1,2]. Bone tumours include more than twenty diagnostic sub-groups and are the third most common cancer diagnosed in 10–24 year olds. The most common sub-group is osteosarcoma which encompasses more than one third of all bone cancers. Osteosarcoma reaches a first prominent incidence peak in late childhood or adolescence around the time of the pubertal growth spurt and then declines. There is a secondary peak in adults aged more than 65 years. Ewing sarcoma peaks in incidence in the late adolescent years [3-6].

A small number of studies have explored geographical patterning in the incidence of bone cancers. These have been focused on paediatric cases, aged 0–14 years [7,8]. One recent analysis of childhood incidence data (aged 0–14 years) from the whole of Great Britain (GB) has found space-time clustering amongst cases of osteosarcoma, but not Ewing sarcoma. Furthermore, space-time clustering was statistically significant for female cases of osteosarcoma, but not for male cases [7]. This was interpreted as providing support for the involvement of a geographically heterogeneous and intermittent environmental exposure in the aetiology of osteosarcoma. Another analysis of the same GB data set found increased risk of childhood Ewing sarcoma in areas of greater socio-economic affluence [8].

A recent review of the literature on childhood bone cancer (specifically osteosarcoma and Ewing sarcoma) has found that several environmental associations were reported consistently. This included an association between parental farming and residence on a farm and higher risk of all bone cancers, especially Ewing sarcoma [9]. Geographical or socio-demographic variation in risk is especially indicative of an environmental component to aetiology, which may also be associated with gene environment interactions.

In light of these previous findings, the aim of the present study was to test predictions of spatial variation occurring among osteosarcoma and Ewing sarcoma that might arise as a result of environmental mechanisms related to area-level population density and area-level socio-economic deprivation. The following aetiological hypotheses were tested: a primary factor influencing geographical heterogeneity of incidence of osteosarcoma or Ewing sarcoma is modulated by differences between environmental exposures occurring in (i) less and more densely populated areas of residence; (ii) less and more socio-economically deprived areas of residence; and geographical heterogeneity of incidence of osteosarcoma or Ewing sarcoma is modulated by (iii) age; and (iv) gender. The analyses extend the upper bound of the age range covered by the previous childhood analyses from 15 to 50 years, thus including the peak ages for occurrence of osteosarcoma and Ewing sarcoma.

## **Methods**

### **Study subjects**

Data were included for all patients who were diagnosed with primary osteosarcoma or Ewing sarcoma in the whole of GB (England, Scotland and Wales) during the period 1^st^ January 1980 to 31^st^ December 2005 and who were aged less than fifty years at the time of diagnosis. Cases were limited to this age range because the incidence of Ewing sarcoma is extremely low for cases above the age of fifty years [1,3] and the second peak of osteosarcoma in the older age group has other possible aetiologies, including as secondary to Paget’s disease and radiotherapy [4,10]. General cancer registration in GB is conducted by eight regional registries in England and by separate Scottish and Welsh registries. Most of the registries get their principal information from hospitals’ patient administration systems (usually electronically) and pathology laboratories. Some of the registries also use hospital records staff to collect data, while others employ peripatetic clerks who visit hospitals. In addition, registries regularly receive notifications of deaths from the Office for National Statistics where cancer is mentioned on the death certificate. Registries match these against their records to indicate possible cases not already known to them, or to update details of existing records. The final datasets of all incident cases of cancer are thereby constructed.

After gaining all necessary regulatory and ethical approvals (UK National Research Ethics Service, Sunderland Research Ethics Committee reference number 09/H0904/5) West Midlands Cancer Intelligence Unit (WMCIU) coordinated the request for data from all regional cancer registries in England and Wales. A separate request was made to the Information Services Division for Scotland in order for data to be released from the Scottish Cancer Registry.

Case data for children, aged 0–14 years, were also extracted from the National Registry of Childhood Tumours (NRCT). The NRCT is a population-based registry covering the whole of GB [11]. It includes records for nearly all children, aged 0–14 years, diagnosed with cancer from 1962 to the present day. This data set was used to cross-check the accuracy of the case counts for childhood data from the regional registries.

### **Diagnostic groups**

Cases were classified into diagnostic groups according to the International Classification of Diseases for Oncology, third edition (ICD-O-3) [12]. All diagnoses were coded to ICD-O-3 using information on pathology (morphology and topography). The following diagnostic groups were specified a priori for analysis: (i) osteosarcoma (ICD-O-3 topography codes for sites classified as bones and joint: C400-C403, C408-C414, C418-419 and associated morphology codes 9180/3; 9181/3, 9182/3, 9183/3, 9184/3, 9185/3, 9186/3, 9187/3, 9192/3, 9193/3, 9194/3, 9195/3) and (ii) Ewing sarcoma (ICD-O-3 topography codes for sites classified as bones and joint: C400-C403, C408-C414; C418-C419, C760-C768 and associated morphology code 9260/3; 9261/3).

### **Population data**

For England and Wales, the sub-national hierarchy of geographies, for which population data are available, is as follows (largest to smallest): government office region (0–49 population ranges from 1,660,000 to 5,600,000, median = 3,430,000); local authority district (0–49 population ranges from 1,200 to 720,900, median = 72,600); and census ward (0–49 population ranges from 297 to 29,300, median = 3,090). In Scotland, postcode sectors are equivalent to census wards (0–49 population ranges from 23 to 15,916, median = 3,201). In this study, analyses were performed at the small-area census ward level in England and Wales and postcode sector level in Scotland. During the study period, there were three censuses in the whole of GB [13-15]. There were also widespread boundary changes throughout this time span, especially at small-area level. To allow for these perturbations, Norman’s method was used to derive population estimates using the small-area boundaries that pertained at the time of the 2001 census [16].

### **Demographic data**

Small-area (census ward in England and Wales and postcode sector in Scotland) demographic characteristics were derived from the censuses [17-19]. These characteristics were population density (persons per hectare) and level of deprivation. The Townsend score for deprivation at the small-area level (and not individual level) was calculated [20]. This is a combination of four census measures: unemployment, household with no car, non-home ownership and household overcrowding. A time series of Townsend deprivation scores was constructed by apportioning these four constituent measures from the 1981, 1991 and 2001 censuses (applied to 1980–1985, 1986–1995 and 1996–2005 data, respectively) to the 2001 census geography [21]. Increasingly negative Townsend scores represent lower area deprivation. Increasingly positive scores represent higher deprivation. Population density was apportioned in a similar way.

### **Statistical methods**

Age-specific incidence rates per million persons per year were calculated based on annual mid-year population estimates for the study region obtained from the Office for National Statistics (ONS). Comparisons of age-standardized incidence rates (ASRs) are only meaningful if they are standardized in a similar fashion. ASRs were calculated using the standard world population (originally proposed by Segi, but modified by Doll and colleagues and constructed from the pooled populations of forty-six representative countries that had accurate population data) [2,22-24]. Temporal trends were assessed using Poisson regression. An assumption of a linear trend was tested by inclusion of a non-linear (categorical) term for year in the model. The interaction between gender and time was also analyzed.

For the ecological analysis, there was evidence of extra-Poisson variation: for osteosarcoma 98.5% of age group and gender specific small-area (wards or postcode sectors) cells had zero counts and for Ewing sarcoma 99.1% of age group and gender specific small-area (wards or postcode sectors) cells had zero counts. Therefore, the incidence of osteosarcoma and Ewing sarcoma was modelled at the census small-area level using negative binomial regression in STATA [25]. The number of cases observed in each small-area was the dependent variable and the logarithm of the underlying population was used as the offset. The ecological (independent) variables were the census-derived small-area characteristics, which were allocated to the 2001 census geography using Norman’s method [21].

A series of multivariable models were fitted including the following independent variables: gender, age (categorized in three groups as: 0–14, 15–29 and 30–49 years), population density, Townsend score (as a composite). The following components of the Townsend score were included in separate models that did not include the composite score: percentage of overcrowded houses, percentage of households without a car, percentage of households with residents unemployed and percentage of homes that are not owner occupied. Interactions between age and gender (age*gender), region and gender (region*gender) and the Townsend deprivation score and gender (Townsend*gender) were also considered for inclusion in the models. Each variable in turn was removed and compared using a likelihood ratio test. Thus, the effect of each variable was assessed by calculating differences in residual deviances and comparing with a chi-square distribution with degrees of freedom (df) equal to the difference in residual degrees of freedom. Model fit was assessed using both the residual deviance and the Akaike information criterion (AIC). Linearity assumptions were tested by including quintiles of significant continuous variables as ordinal variables in the models.

Significant effects are reported as relative risks (RRs) and associated 95% confidence intervals (CIs). All *P* values were two-sided and statistical significance was taken as *P* < 0.05 throughout the analyses.

## **Results**

### **Osteosarcoma**

There were a total of 2566 patients aged 0–49 years (1493 males and 1073 females) diagnosed with osteosarcoma in GB between 1980 and 2005. The ASR over the study period was 2.64 per million persons per year (95% CI 2.53 to 2.74) for all 0–49 year olds. For males and females, the overall ASRs were 3.00 (95% CI 2.85 to 3.16) and 2.27 (95% CI 2.13 to 2.41) per million persons per year, respectively. Case numbers, crude rates and ASRs by age-group, period, region and gender are given in Table 1. An assumption of a linear trend was confirmed to be valid and Poisson regression showed that there was a significant annual increase in the incidence of osteosarcoma of 1.0% (95% CI 0.5 to 1.5) over the study period. The ratio of the ASRs for males: females was 1.3 (95% CI 1.2 to 1.4) and this remained constant over the study period (*P* = 0.415).

**Table 1:** Rates of Osteosarcoma in GB by age, period, region and gender during 1980-2005

	**N**	**Pop (000’s)**	**All Crude Rate / million**	**ASR (95% CI)**	**N**	**Pop (000’s)**	**Male Crude Rate / million**	**ASR (95% CI)**	**N**	**Pop (000’s)**	**Female Crude Rate / million**	**ASR (95 % CI)**	**ASR Ratio M:F (95% CI)**
Age													
Ages 0 to 14	817	279909	2.92	2.78 (2.59,2.97)	406	142623	2.85	2.70 (2.44,2.97)	411	137286	2.99	2.86 (2.58,3.14)	0.9 (0.8,1.1)
Ages 15 to 29	1315	311514	4.22	3.97 (3.76,4.19)	821	156621	5.24	4.95 (4.61,5.29)	494	154894	3.19	2.98 (2.71,3.24)	1.7 (1.5,1.9)
Ages 30 to 49	434	400790	1.08	1.08 (0.97,1.18)	266	199591	1.33	1.33 (1.17,1.49)	168	201199	0.83	0.83 (0.70,0.95)	1.6 (1.3,1.9)
Period													
1980 - 1985	524	225881	2.32	2.15 (1.96,2.34)	304	113741	2.67	2.44 (2.17,2.72)	220	112140	1.96	1.85 (1.60,2.10)	1.3 (1.1,1.6)
1986 - 1995	1010	382985	2.64	2.74 (2.57,2.91)	607	192375	3.16	3.21 (2.95,3.47)	403	190609	2.11	2.26 (2.04,2.49)	1.4 (1.2,1.6)
1996 - 2005	1032	383347	2.69	2.84 (2.67,3.02)	582	192718	3.02	3.15 (2.89,3.41)	450	190629	2.36	2.53 (2.29,2.77)	1.2 (1.1,1.4)
Region													
North East	123	45292	2.72	2.75 (2.25,3.24)	73	22711	3.21	3.18 (2.44,3.92)	50	22581	2.21	2.31 (1.66,2.96)	1.4 (0.9,1.9)
North West	278	120449	2.31	2.31 (2.03,2.58)	168	60464	2.78	2.73 (2.32,3.15)	110	59985	1.83	1.88 (1.53,2.24)	1.5 (1.1,1.8)
Yorkshire & Humber	236	87272	2.70	2.71 (2.36,3.06)	147	43870	3.35	3.32 (2.78,3.86)	89	43403	2.05	2.09 (1.65,2.53)	1.6 (1.2,2.0)
East Midlands	193	71476	2.70	2.74 (2.34,3.13)	100	36038	2.77	2.78 (2.23,3.33)	93	35438	2.62	2.69 (2.13,3.25)	1.0 (0.7,1.3)
West Midlands	238	93160	2.55	2.59 (2.25,2.92)	128	46964	2.73	2.68 (2.21,3.15)	110	46196	2.38	2.49 (2.02,2.96)	1.1 (0.8,1.4)
East of England	233	91147	2.56	2.64 (2.30,2.99)	129	45929	2.81	2.87 (2.37,3.37)	104	45218	2.30	2.41 (1.94,2.88)	1.2 (0.9,1.5)
London	341	129437	2.63	2.88 (2.56,3.20)	200	64819	3.09	3.31 (2.83,3.78)	141	64618	2.18	2.46 (2.04,2.88)	1.3 (1.0,1.6)
South East	353	135307	2.61	2.67 (2.39,2.95)	197	68226	2.89	2.91 (2.50,3.32)	156	67081	2.33	2.42 (2.04,2.81)	1.2 (0.9,1.5)
South West	198	79244	2.50	2.52 (2.16,2.87)	115	39917	2.88	2.83 (2.30,3.35)	83	39327	2.11	2.20 (1.72,2.69)	1.3 (0.9,1.7)
Wales	119	49236	2.42	2.37 (1.94,2.80)	77	24700	3.12	3.01 (2.33,3.69)	42	24536	1.71	1.72 (1.19,2.25)	1.7 (1.1,2.4)
Scotland	228	90201	2.53	2.55 (2.22,2.89)	144	45202	3.19	3.19 (2.66,3.72)	84	44999	1.87	1.90 (1.48,2.31)	1.7 (1.2,2.1)
**Total**^**1**^	**2566**	**992213**	**2.59**	**2.64 (2.53,2.74)**	**1493**	**498835**	**2.99**	**3.00 (2.85,3.16)**	**1073**	**493379**	**2.17**	**2.27 (2.13,2.41)**	**1.3 (1.2,1.4)**

^*1*^*Includes 26 cases with unknown region.*

The analyses of deviance and AIC showed that model fit for osteosarcoma was significantly improved for both gender (*P* < 0.0001) and age (*P* < 0.0001) and that there was a significant interaction between gender and age (*P* < 0.0001), with lower female rates overall, but higher rates in females aged 0–14 years. There was, however, no significant variation in incidence between geographical regions (*P* = 0.8346), nor was there any interaction between gender and region (*P* = 0.334). Townsend score (as a composite), and then in separate models with all four of its component variables, were statistically significant (for overall Townsend score: *P* < 0.0001) compared with the model containing age, gender and age*gender. Furthermore, there was a statistically significant interaction between Townsend score and gender (*P* = 0.0102). Population density was associated with a statistically significant improvement in model fit (*P* = 0.0001) compared with the model containing age, gender and age*gender, but was not significant when compared with the model containing age, gender, age*gender and Townsend score (*P* = 0.1388). The best fitting model contained: gender, age, the interaction gender*age, the Townsend score and the interaction Townsend*gender. Table 2 gives a comparison of the goodness-of-fit of the different models, assessed using residual deviance and AIC with model 17 denoting the best-fitting model. An assumption of a linear trend for the Townsend score was confirmed (*P* < 0.001).

**Table 2:** Hierarchical series of models for Osteosarcoma with goodness of fit diagnostics

**Model**	**Factors/interactions**	**df**^**1**^	**deviance**	**AIC**^**2**^
0	Null	175672	18788.0	25587.9
1	Gender	175671	18728.0	25529.9
2	Age	175670	18074.1	24878.0
3	Age & Gender	175669	18017.2	24823.1
4	Age, Gender*Age	175668	17976.3	24784.2
5	Age, Gender*Age & Region	175658	17970.6	24798.5
6	Age, Gender*Age & Population density	175667	17961.4	24771.3
7	Age, Gender*Age & Townsend	175667	17947.5	24757.4
8	Age, Gender*Age & Overcrowding	175667	17968.4	24778.3
9	Age, Gender*Age & No cars	175667	17951.4	24761.3
10	Age, Gender*Age & Unemployment	175667	17955.9	24765.8
11	Age, Gender*Age & Home ownership	175667	17956.9	24766.8
12	Age, Gender*Age, Townsend & Population density	175666	17945.3	24757.2
13	Age, Gender*Age, Overcrowding & Population density	175666	17945.2	24757.1
14	Age, Gender*Age, No cars & Population density	175666	17947.5	24759.4
15	Age, Gender*Age, Unemployment & Population density	175666	17947.5	24759.4
16	Age, Gender*Age, Home ownership & Population density	175666	17947.1	24759.0
17	Age, Gender*Age, Townsend & Townsend*female	175666	17940.9	24752.8

^*1*^*df = residual degrees of freedom,*^*2*^*AIC = Akaike Information Criterion.*

Table 3 gives the RRs for the best fitting model containing gender, age, the interaction gender*age, the Townsend score and the interaction Townsend*gender. This shows that the protective effect of increased levels of deprivation is greater for females than for males. For females, a statistically significant decreased risk was associated with higher Townsend score (i.e. more deprived, RR for one unit increase in the deprivation score = 0.969; 95% CI 0.946 to 0.993).

**Table 3:** Effect on Osteosarcoma incidence of gender (female), age and Townsend deprivation score

**Factor/interaction**	**Coefficient (95% CI^1^)**	**RR^2^ (95% CI)**	***P* value**
Female	−0.490 (−0.588,-0.391)	0.613 (0.555,0.676)	<0.001
Age 15-29	0.648 (0.528,0.768)	1.912 (1.696,2.155)	<0.001
Age 30-49	−0.739 (−0.882,-0.597)	0.477 (0.414,0.551)	<0.001
Female & age 0-14	0.551 (0.380,0.722)	1.736 (1.463,2.059)	<0.001
Townsend	−0.019 (−0.035,-0.004)	0.981 (0.966,0.996)	0.015
Female & Townsend	−0.032 (−0.056,-0.007)	0.969 (0.946,0.993)	0.010

^*1*^*CI = Confidence Interval,*^*2*^*RR = Relative Risk.*

### **Ewing sarcoma**

There were a total of 1650 patients aged 0–49 years (988 males and 662 females) diagnosed with Ewing sarcoma in GB between 1980 and 2005. The ASR over the study period was 1.76 per million persons per year (95% CI 1.67 to 1.84) for all 0–49 year olds. For males and females, the overall ASRs were 2.06 (95% CI 1.92 to 2.19) and 1.45 (95% CI 1.34 to 1.56) per million persons per year, respectively. Case numbers, crude rates and ASRs by age-group, period, region and gender are given in Table 4. Poisson regression showed a significant annual increase in the incidence of Ewing sarcoma of 1.2% (95% CI 0.6-1.9) over the study period. The ratio of the ASRs for males: females was 1.4 (95% CI 1.3 to 1.6) and this remained constant over the study period (*P* = 0.123).

**Table 4:** Rates of Ewing Sarcoma in GB by age, period, region and gender during 1980-2005

	**N**	**Pop (000’s)**	**All Crude Rate / million**	**ASR (95% CI)**	**N**	**Pop (000’s)**	**Male Crude Rate/ million**	**ASR (95% CI)**	**N**	**Pop (000’s)**	**Female Crude Rate/ million**	**ASR (95% CI)**	**ASR Ratio M:F (95% CI)**
Age													
Ages 0 to 14	659	279909	2.35	2.28 (2.11,2.46)	356	142623	2.50	2.43 (2.17,2.68)	303	137286	2.21	2.13 (1.89,2.37)	1.1 (1.0,1.3)
Ages 15 to 29	800	311514	2.57	2.39 (2.23,2.56)	516	156621	3.29	3.06 (2.79,3.32)	284	154894	1.83	1.71 (1.51,1.92)	1.8 (1.5,2.0)
Ages 30 to 49	191	400790	0.48	0.47 (0.40,0.53)	116	199591	0.58	0.57 (0.47,0.67)	75	201199	0.37	0.36 (0.28,0.45)	1.6 (1.1,2.0)
Period													
1980 - 1985	331	225881	1.47	1.46 (1.30,1.62)	186	113741	1.64	1.58 (1.35,1.82)	145	112140	1.29	1.33 (1.11,1.55)	1.2 (0.9,1.5)
1986 - 1995	649	382985	1.69	1.81 (1.67,1.95)	393	192375	2.04	2.16 (1.94,2.37)	256	190609	1.34	1.46 (1.28,1.64)	1.5 (1.2,1.7)
1996 - 2005	670	383347	1.75	1.90 (1.76,2.05)	409	192718	2.12	2.26 (2.04,2.48)	261	190629	1.37	1.53 (1.34,1.72)	1.5 (1.2,1.7)
Region													
North East	69	45292	1.52	1.64 (1.25,2.03)	44	22711	1.94	2.08 (1.46,2.70)	25	22581	1.11	1.19 (0.77,1.76)	1.7 (0.9,2.6)
North West	191	120449	1.59	1.61 (1.38,1.84)	112	60464	1.85	1.86 (1.51,2.21)	79	59985	1.32	1.37 (1.06,1.67)	1.4 (1.0,1.8)
Yorkshire & Humber	152	87272	1.74	1.83 (1.53,2.12)	93	43870	2.12	2.18 (1.73,2.63)	59	43403	1.36	1.47 (1.09,1.85)	1.5 (1.0,2.0)
East Midlands	140	71476	1.96	2.02 (1.68,2.36)	68	36038	1.89	1.90 (1.44,2.36)	72	35438	2.03	2.14 (1.64,2.64)	0.9 (0.6,1.2)
West Midlands	130	93160	1.40	1.45 (1.20,1.70)	77	46964	1.64	1.70 (1.32,2.09)	53	46196	1.15	1.19 (0.86,1.52)	1.4 (0.9,1.9)
East of England	141	91147	1.55	1.68 (1.40,1.97)	85	45929	1.85	1.98 (1.55,2.40)	56	45218	1.24	1.39 (1.02,1.75)	1.4 (0.9,1.9)
London	181	129437	1.40	1.58 (1.34,1.81)	114	64819	1.76	1.97 (1.60,2.34)	67	64618	1.04	1.18 (0.88,1.47)	1.7 (1.1,2.2)
South East	212	135307	1.57	1.70 (1.46,1.93)	120	68226	1.76	1.84 (1.50,2.17)	92	67081	1.37	1.55 (1.23,1.87)	1.2 (0.9,1.5)
South West	141	79244	1.78	1.86 (1.55,2.18)	93	39917	2.33	2.39 (1.90,2.89)	48	39327	1.22	1.31 (0.93,1.69)	1.8 (1.2,2.5)
Wales	85	49236	1.73	1.80 (1.41,2.18)	57	24700	2.31	2.35 (1.74,2.97)	28	24536	1.14	1.23 (0.81,1.79)	1.9 (1.0,2.8)
Scotland	197	90201	2.18	2.25 (1.93,2.57)	119	45202	2.63	2.68 (2.19,3.17)	78	44999	1.73	1.81 (1.40,2.22)	1.5 (1.1,1.9)
**Total**^**1**^	**1650**	**992213**	**1.66**	**1.76 (1.67,1.84)**	**988**	**498835**	**1.98**	**2.06 (1.92,2.19)**	**662**	**493379**	**1.34**	**1.45 (1.34,1.56)**	**1.4 (1.3,1.6)**

^*1*^*Includes 11 cases with unknown region.*

The analyses of deviance and AIC show that incidence of Ewing sarcoma was associated with both gender (*P* < 0.0001) and age (*P* < 0.0001). There was also an interaction between gender and age (*P* < 0.0001), with lower female rates overall, but higher rates in females aged 0–14 years. Incidence varied by region (*P* < 0.0001). However, further improvement to model fit (AIC) was provided by inclusion of covariates for both East Midlands and Scotland in the model (Table 5, model 7). Incidence was higher in the East Midlands (RR = 1.207; 95% CI 1.012 to 1.440; *P* = 0.0356) and Scotland (RR = 1.428; 95% CI 1.221 to 1.671; *P* < 0.001) (Table 5). However, there was no interaction between gender and region (*P* = 0.621).

**Table 5:** Hierarchical series of models for Ewing Sarcoma with goodness of fit diagnostics

**Model**	**Factors/interactions**	**df**^**1**^	**deviance**	**AIC**^**2**^
0	Null	175672	13680.3	18089.1
1	Gender	175671	13622.2	18033.0
2	Age	175670	13015.2	17428.0
3	Age & Gender	175669	12960.6	17375.3
4	Age, Gender, Age*Gender	175668	12942.9	17359.7
5	Age, Gender, Age*Gender & Region	175658	12911.1	17347.9
6	Age, Gender, Age*Gender, EM^3^	175667	12938.4	17357.2
7	Age, Gender, Age*Gender, Scotland, EM	175666	12921.3	17342.1
8	Age, Gender, Age*Gender, Scotland, EM & Population density	175665	12883.6	17306.4
9	Age, Gender, Age*Gender, Scotland, EM & Townsend	175665	12895.5	17318.2
10	Age, Gender, Age*Gender, Scotland, EM & Overcrowding	175665	12912.1	17334.9
11	Age, Gender, Age*Gender, Scotland, EM & No cars	175665	12888.6	17311.4
12	Age, Gender, Age*Gender, Scotland, EM & Unemployment	175665	12904.7	17327.5
13	Age, Gender, Age*Gender, Scotland, EM & Home ownership	175665	12906.8	17329.6
14	Age, Gender, Age*Gender, Scotland, EM, Population density & No cars	175664	12875.2	17300.0
15	Age, Gender, Age*Gender, Scotland, EM, Pop dens & Unemployment	175664	12878.4	17303.2
16	Age, Gender, Age*Gender, Scotland, EM, Population density & Townsend	175664	12878.0	17302.8
17	Age, Gender, Age*Gender, Scotland, EM, Pop dens & Overcrowding	175664	12883.4	17308.2
18	Age, Gender, Age*Gender, Scotland, EM, Pop dens & Home owner	175664	12881.5	17306.3

^*1*^*df = residual degrees of freedom,*^*2*^*AIC = Akaike Information Criterion,*^*3*^*East Midlands.*

Population density was statistically significant (*P* < 0.0001), compared with the model containing gender, age, the interaction gender*age, East Midlands and Scotland. Townsend score (as a composite) and then in separate models with all of its components (non-car ownership, unemployment, housing tenure and household overcrowding) was statistically significant (for overall Townsend score: *P* < 0.0001), compared with the model containing gender, age, the interaction gender*age, East Midlands and Scotland. Furthermore, Townsend score (as a composite), and then in separate models containing two of its components (non-car ownership and unemployment, but not housing tenure and household overcrowding) remained statistically significant (for overall Townsend score: *P* = 0.0181), compared with the model containing gender, age, gender*age, East Midlands, Scotland and population density. However, the best fitting model contained: age, gender, age*gender, East Midlands, Scotland, population density and non-car ownership. Table 5 gives a comparison of the goodness-of-fit of the different models, assessed using residual deviance and AIC with model 14 denoting the best-fitting model. Assumptions of linear trends for population density and non-car ownership were confirmed (*P* < 0.001 for both variables).

Table 6 gives the RRs for the best fitting model containing age, gender, age*gender, population density and non-car ownership. This shows that statistically significant decreased risk was associated with greater area-level population density (RR for an increase of one person per hectare = 0.984 95% CI 0.976 to 0.993) and was also associated with greater levels of non-car ownership (RR for an increase of one percent in non-car ownership = 0.994; 95% CI 0.991 to 0.998).

**Table 6:** Effect on Ewing Sarcoma incidence of female gender, age, East Midlands, Scotland, population density and non-car ownership

**Factor/interaction**	**Coefficient (95% CI^1^)**	**RR^2^ (95% CI)**	***P* value**
Female	−0.548 (−0.679,-0.417)	0.578 (0.507,0.659)	<0.001
Age 15-29	0.316 (0.180,0.452)	1.372 (1.198,1.571)	<0.001
Age 30-49	−1.385 (−1.569,-1.201)	0.250 (0.208,0.301)	<0.001
Female & age 0-14	0.433 (0.230,0.636)	1.542 (1.259,1.890)	<0.001
East Midlands	0.188 (0.012,0.364)	1.207 (1.012,1.440)	0.037
Scotland	0.356 (0.199,0.513)	1.428 (1.221,1.671)	<0.001
Pop density (person / hectare)	−0.016 (−0.025,-0.007)	0.984 (0.976,0.993)	<0.001
Non-car ownership (%)	−0.006 (−0.009,-0.002)	0.994 (0.991,0.998)	0.004

^*1*^*CI = Confidence Interval,*^*2*^*RR = Relative Risk.*

## **Discussion**

This is the first comprehensive small-area analysis of osteosarcoma and Ewing sarcoma in 0–49 year olds from GB. Furthermore, it is the largest geographical study of these tumour types to date. GB is an ideal setting for this type of investigation due to the availability of highly accurate and complete cancer registration data, together with corresponding population census data. The study has revealed two novel findings: (a) for females lower incidence of osteosarcoma was associated with higher levels of deprivation; (b) lower incidence of Ewing sarcoma was associated with residence in more densely populated areas and higher levels of non-car ownership.

Our prior hypotheses were: a primary factor influencing geographical heterogeneity of incidence of osteosarcoma or Ewing sarcoma is modulated by differences between environmental exposures occurring in (i) less and more densely populated areas of residence; (ii) less and more socio-economically deprived areas of residence; and geographical heterogeneity of incidence of osteosarcoma or Ewing sarcoma is modulated by (iii) age; and (iv) gender.

For osteosarcoma the results suggest that, at least for females, geographical heterogeneity of incidence is modulated by differences in environmental exposures occurring in less and more socio-economically deprived areas of residence (providing support for prior hypotheses (ii) and (iv), but little support for prior hypotheses (i) and (iii)). For Ewing sarcoma the results suggest that geographical heterogeneity of incidence is modulated by differences in environmental exposures occurring in less and more densely populated areas and that incidence is also modulated by some aspect of differences between environmental exposures occurring in less and more socio-economically deprived areas of residence. However, some of the components of deprivation may be confounded with population density apart from non-car ownership (providing support for prior hypotheses (i) and (ii)). Comparison of the case counts for both osteosarcoma and Ewing sarcoma from the WMCIU for children aged 0–14 years across all regions confirmed virtually identical agreement with the counts from the NRCT dataset.

Two methodological caveats should be noted. First of all, census ward (or postcode sector) population density and Townsend deprivation scores are not necessarily related to characteristics of individual cases and should only be regarded as ecological measurements. Area-level data have been assigned to individual cases. Care should be exercised when using such grouped data to make inferences about individuals. There may be unknown confounding factors that display the same pattern of spatial heterogeneity [26]. Secondly, the case, population and demographic data were analyzed using 2001 census boundaries. The method did not take into account the possible effects of migration, which may have diluted the results. Nevertheless, the findings were very clear cut, indicating that this does not appear to have been a major limitation.

The study has a number of particular strengths. First, it analyses high-quality population-based data. Secondly, the inclusion of ages 0 to 49 years includes the peak incidence of both osteosarcoma and Ewing sarcoma, which occur in the teenage years. There is a theoretical possibility that diagnosis delays vary according to some of the demographic factors that have been analyzed here. Consequently, a lower maximum age limit might have led to differential loss of some cases, according to demographics.

Previous studies have explored geographical patterning in the incidence of bone cancers, but have been focused on children, aged 0–14 years [7,8]. An analysis of childhood incidence data in GB found space-time clustering amongst cases of osteosarcoma, but not Ewing sarcoma. The space-time clustering was significant for females, but not for males [7]. This was interpreted as providing support for the involvement of a geographically heterogeneous and transient environmental exposure in the aetiology of osteosarcoma. Effects of such an exposure may be modulated by gender. Another analysis of the same national GB data set found increased risk of childhood Ewing sarcoma in areas of greater socio-economic affluence [8]. However, the age-specific incidence of these tumours means that to cut off analyses at age 14 years is artefactual and an extended age range to cover the majority of the conditions is more appropriate, such as the one described here. It is possible that the splitting of age–groups at 0–14 and 15–29 years may have led to a dilution of the number of male cases in the peak age-range which straddle both groups and thus have led to the female-specific effect for deprivation amongst cases of osteosarcoma. However, a further supplementary analysis used age groups 0–29 and 30–49 years and found that the interaction between deprivation and gender was still present (data not shown).

The aetiology of osteosarcoma is likely to involve both genetic predisposition and environmental triggers. However, known genetic factors only account for a small proportion of cases [9,27-33]. The present study has shown that increased levels of deprivation (i.e. less affluence) are protective against osteosarcoma. This suggests that differences in some aspect of lifestyle may predispose to greater risk of osteosarcoma. These differences may include both dietary and social factors. A meta-analysis of fourteen studies has found that the mean height of osteosarcoma patients was two to three centimetres greater than the reference population. The authors described this finding as a surrogate for affluence. However, there was no obvious bias towards females [34]. Another pooled analysis (of seven studies) found that taller than average individuals had increased risk of osteosarcoma and very tall individuals had even greater risk [35]. Such differences in height are characteristic of more affluent living conditions during childhood. A further recent descriptive analysis of incident data on cases of bone cancer, diagnosed in England during the period 1979–2003 showed that the female peak was earlier (10–14 years) than for males (15–19 years). The authors proposed that pubertal bone growth may be implicated [36]. Our findings suggest that during this period females may be more vulnerable to a putative environmental hazard as a consequence of hormonal effects. Another small case–control study of juvenile bone tumours (in 88 patients aged 8–25 years) found increased risk associated with frequent change of residence and previous mumps [37]. Together with finding of space–time clustering, this suggests that it is possible that one or more infectious agents may be implicated [7]. The possible association with frequent change of residence would suggest that some aspect of population mixing may be implicated. We would postulate that the pathway is likely to be indirect. Such a mechanism has been proposed for childhood leukaemia, with unusual population mixing or delayed exposure to common infections in early life conferring greater risk [38]. Further epidemiological studies (e.g. using a case–control design) are needed to determine if infections occurred more frequently prior to diagnosis in cases of osteosarcoma, compared with an unaffected control group. The nature and type of putative infections should also be investigated. However, it should be noted that the rarity of this condition would make the conducting of a case–control study relatively expensive.

Both genetic and environmental factors are also implicated in the aetiology of Ewing sarcoma. However, again genetic factors alone can only explain a small fraction of the total cases [39,40]. The present study has shown a higher risk with residential living in less densely populated areas, but also with higher levels of car ownership. These are both characteristic of rural areas. Markedly higher incidence was apparent in East Midlands and Scotland, which both contain large areas of rural expanse. Rural living, together with high levels of car ownership, may also be consistent with a certain type of socio-economic affluence. Potential exposures that have been linked with Ewing sarcoma include both pesticides and zoonotic infectious agents [41-43]. If infectious agents are involved the mechanism is likely to be different from osteosarcoma, as Ewing sarcoma did not exhibit space-time clustering [7]. Further studies should determine if higher risk of Ewing sarcoma is associated with residential living in close proximity to areas with predominantly agricultural land use. Analyses could include investigating possible associations with land use data, including types of crop grown and pesticides used.

## **Conclusions**

We have found that lower incidence of osteosarcoma in females was observed in areas associated with higher levels of deprivation indicating decreased risk is linked to some aspect of less affluent living conditions. Lower incidence of Ewing sarcoma occurred in areas of high population density and where fewer people owned cars, suggesting rural area characteristics are linked to increased risk of this malignancy. The study adds substantially to the growing body of evidence that associates risk of Ewing sarcoma with rural environmental exposures. Putative risk factors include agricultural exposures, such as pesticides and zoonotic agents.

## **Competing interests**

The author(s) declare that they have no competing interests.

## **Authors’ contributions**

RJQM, KB, RCP, PWJ, PN, AWC and RGF contributed to the design of the study, the writing of the manuscript and the analysis and interpretation of data. BGP, CS, TJV, PAM and MFM contributed to the writing of the manuscript and the interpretation of data. All authors read and approved the final manuscript.

## Pre-publication history

The pre-publication history for this paper can be accessed here:

http://www.biomedcentral.com/1471-2407/12/270/prepub
